# Pediatric idiopathic iliac artery aneurysm repair with limited conduit options

**DOI:** 10.1177/17085381251379286

**Published:** 2025-09-11

**Authors:** David N DeVaro, Alexander S Fairman

**Affiliations:** 1 Department of Vascular Surgery, 6572 University of Pennsylvania, Philadelphia, PA, USA; 2 Department of General, Thoracic, and Fetal Surgery, The Children’s Hospital of Philadelphia, Philadelphia, PA, USA

**Keywords:** Iliac aneurysm, congenital aneurysm, idiopathic aneurysm, child, pediatrics, bypass graft

## Abstract

**Objectives:**

Pediatric arterial aneurysms are very uncommon. Those without an underlying identifiable etiology such as infection or autoimmune disease are even rarer. In young children, options for repair are limited. We report the case of a pediatric common iliac artery aneurysm that was surgically repaired.

**Methods:**

A 27-month-old male presenting for an evaluation of persistent hypertension underwent an abdominal CT scan and was incidentally found to have a large right common iliac artery aneurysm along with a thrombosed, proximal right internal iliac artery aneurysm. Given the size, the aneurysm was repaired with a bypass constructed from cryopreserved femoral artery allograft.

**Results:**

The procedure was uncomplicated, with continued patency of the graft determined via ultrasound at 7 months.

**Conclusion:**

A pediatric iliac artery aneurysm was successfully repaired with a cadaveric femoral artery graft in the setting of limited conduit options.

## Introduction

Congenital iliac artery aneurysms have been rarely reported in the literature. Based on a prior autopsy study, the incidence of isolated congenital iliac aneurysms, that is, without involvement of the aorta, has been estimated at 0.3%.^1^ We are aware of a total of 17 cases of isolated congenital iliac artery aneurysms discovered in the pediatric population.^2–4^ Diagnostic workup typically involves excluding other causes of aneurysm formation, such as rheumatologic or infectious causes. Presenting symptoms have included abdominal pain, a pulsatile abdominal mass, limb length discrepancy, and hydroureteronephrosis.^2^ Surgical management is generally recommended to avoid downstream complications such as aneurysmal rupture and thromboembolism. Management strategies have included both synthetic and autologous bypass grafts, aneurysmorrhaphy, and coil embolization.^2^ Limited ideal conduit options exist in the very young. There are few, if any, reported cases of a congenital iliac aneurysm repaired with femoral artery cadaveric homograft in the pediatric population. Written consent was obtained from the patient’s parents for the publication of this case.

## Case

**Figure 1.**
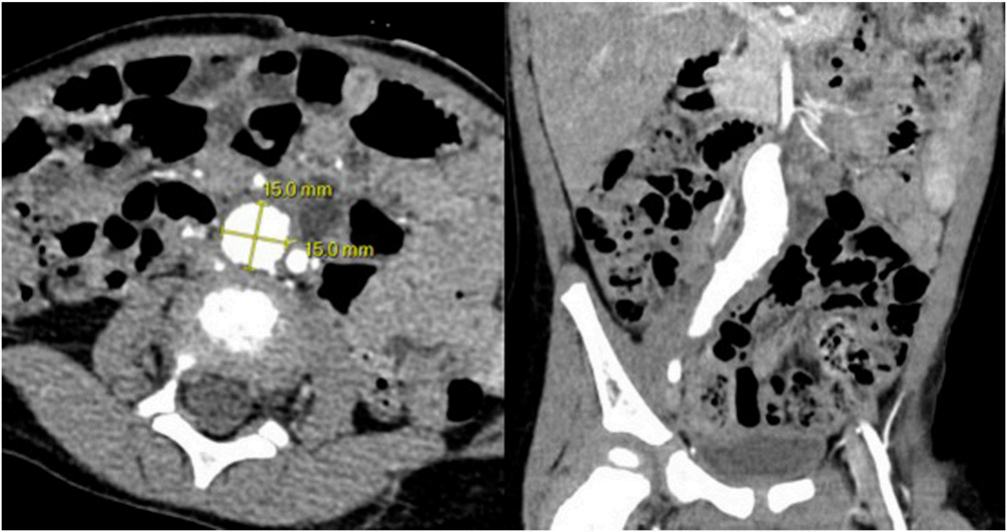
Axial and coronal CT sections demonstrating a 1.5 × 1.5 cm right common iliac artery aneurysm.

The patient was a 27-month-old ex-full term male who was found to have a right common iliac artery aneurysm incidentally on a workup for hypertension. He was first noted to be hypertensive 2 months prior to presentation with systolic blood pressures persistently elevated from the 150s to 190s. CT abdomen and pelvis was performed and was notable for a partially thrombosed aneurysm of the lower pole branch of the right renal artery—a possible cause of his hypertension. It also revealed a 1.5 × 1.5 cm aneurysm of the right common iliac artery from just below the aortic bifurcation to the iliac bifurcation along with an 8 × 8 mm completely thrombosed aneurysm of the right internal iliac artery (Figure 1). No abnormalities were observed in the aorta or the left iliac arteries. An echocardiogram was also performed during the initial workup, notable for mild aortic root dilation.

A rheumatologic workup—including erythrocyte sedimentation rate, C-reactive protein, antinuclear antibodies, lupus anticoagulant, anticardiolipin antibodies, beta-2 glycoprotein antibodies, and an antineutrophil cytoplasmic antibodies panel—was negative. An MRA head and neck was also unremarkable. Given normal inflammatory markers and lack of fevers, an infectious etiology was deemed unlikely. There were no stigmata of tuberous sclerosis or neurofibromatosis on exam. There were also no physical exam findings to support a connective tissue disorder. Family history was negative for these genetic disorders. Given the size of the aneurysm (greater than three times the normal size in that age cohort) and the lack of any baseline imaging to evaluate its growth rate, surgical treatment was planned.

**Figure 2.**
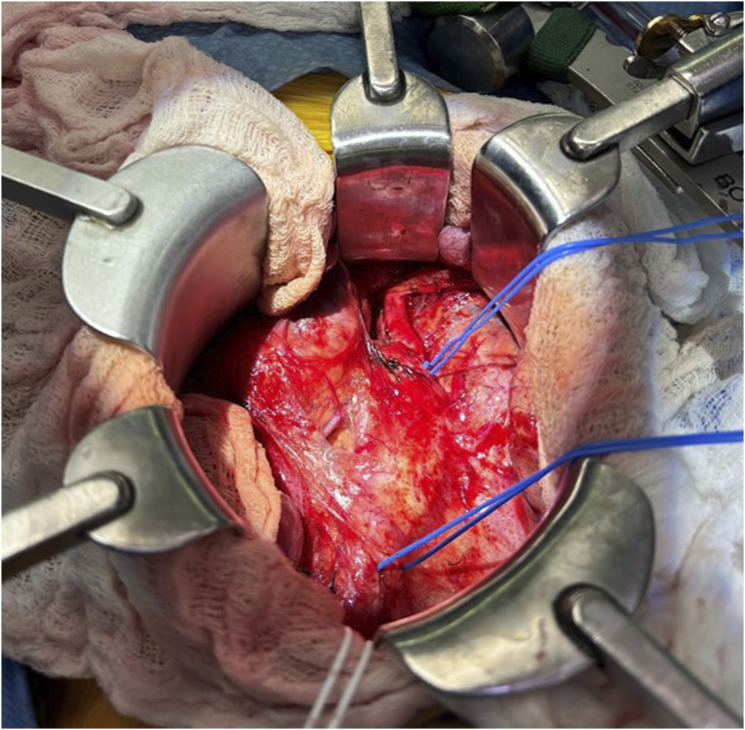
Intraoperative photograph of the right common iliac artery aneurysm prior to resection. Blue vessel loops are placed on the left common iliac artery at the top and the right external iliac artery on the bottom.

**Figure 3.**
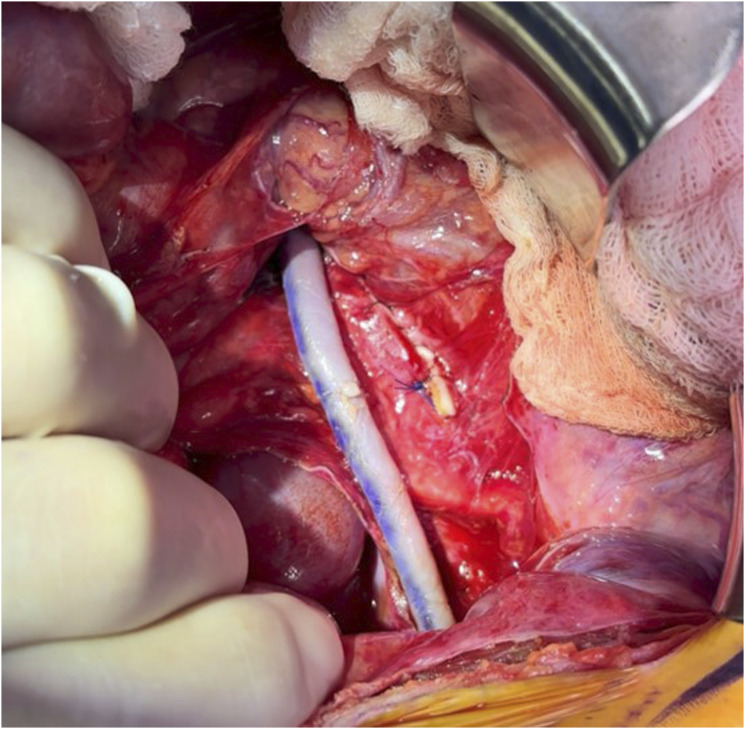
Intraoperative photograph displaying the cadaveric femoral artery bypass in place.

The iliac vessels and aorta were exposed through a lower midline incision (Figure 2). The right common iliac artery was suture ligated proximal to the aneurysm as there was not enough healthy proximal artery available for an end-to-end anastomosis. The prepared cadaveric femoral artery (CryoArtery, Artivion Inc; Kennesaw, Georgia) was spatulated and sewn in an end-to-side fashion above the aortic bifurcation. The internal iliac artery was then suture ligated distal to the thrombosed aneurysm to prevent continued pressurization. The external iliac artery was divided, and the bypass was sewn in an end-to-end fashion using interrupted technique (Figure 3). Care was taken to leave a slight redundancy in the bypass to account for axial growth of the patient over time. There were normal flows confirmed with a sterile Doppler, and the patient had a bounding femoral arterial pulse. A portion of the aneurysmal iliac artery was sent to pathology for analysis.

Histopathologic analysis demonstrated mural fibrosis with some adventitial calcifications and a partially mineralized fibrin clot, without medial degeneration or disruption of elastic fibers. There was minimal lymphocytic inflammation observed. The findings were consistent with a true aneurysm. An institution-specific connective tissue disorder panel was obtained but was only positive for a variant of uncertain significance in *NOTCH1*, which the genetics team did not consider to be a sufficient explanation for his presentation, although further evaluation with whole exome sequencing is planned.

The postoperative course was uneventful. The patient was placed on a heparin drip at 10 units/kg/hr for 3 days and started on aspirin postoperatively the day of the surgery. An ultrasound performed 11 days after the operation prior to discharge showed a patent bypass without stenosis. Continued patency was demonstrated at a 1-month follow-up visit, at 3 months during a procedure by interventional radiology to perform a chemical ablation of a branch of his right renal artery, and at 7 months via ultrasound during another follow-up visit.

## Discussion

Sarkar et al. described nine classes of pediatric aneurysms based on etiology and histopathologic findings. Classification considered the presence of associated infection, autoimmune disease, connective tissue disease, medial degeneration, and vascular dysplasia. Congenital/idiopathic and false aneurysms represented classes VIII and IX, respectively.^5^

In this case, the pathology report was most consistent with an aneurysm of the congenital/idiopathic class, given the reported mural fibrosis without medial degeneration, inflammatory infiltrate, or disruption of vessel layer organization.^5^ Interestingly, of the 17 cases of congenital iliac aneurysms, two were associated with a renal artery aneurysm, as was found here.^3^ The presence of concomitant aortic root disease in this patient is also a notable finding, which appropriately raised suspicion for an underlying connective tissue disorder. Although the panel returned positive for a mutation in *NOTCH1*, *NOTCH1* mutations have not previously been associated with peripheral artery aneurysms.

Chithra et al. suggested operative intervention if the aneurysm exceeded three times the size of the normal adjacent artery.^6^ The mean common iliac artery diameter for children aged 12–36 months has since been estimated at 4.5 mm.^7^ Since the size of the aneurysm exceeded three times this value, surgical management was indicated. A transperitoneal approach was chosen to obtain optimal exposure and vessel control. Simple interrupted sutures were utilized to prevent stenosis due to purse-stringing effects with growth.^6,8^

The decision to use cadaveric femoral artery was on the basis of limited conduit options. Given the size of the patient’s aneurysm, excision with primary repair or aneurysmorrhaphy was not possible. The thrombosed internal iliac aneurysm precluded use of the internal iliac artery as a conduit. Given the patient’s age, the great saphenous veins were diminutive (less than 2 mm in diameter) and inappropriate as a graft. A synthetic graft was avoided due to the patient’s age as well, given concerns for size mismatch with growth.^2,6^ An endovascular approach with a stent graft would be off-label and likely require further intervention as the patient grows. A cadaveric femoral artery graft was deemed preferable over autologous femoral vein and cryopreserved vein due to concerns for chronic venous insufficiency and poor long-term viability in the adult literature, respectively.^9,10^

It is planned for the patient to continue on aspirin for life to reduce the risk of graft occlusion. There is limited data to support the use of life-long aspirin in the pediatric population. Any antiplatelet recommendations are extrapolated from the adult literature. However, given the expected longevity of multiple decades of this patient, we feel that life-long aspirin is prudent to combat intimal hyperplasia over time.

Another follow-up visit is planned for 1 year, with annual ultrasounds thereafter. To monitor for the development of additional aneurysms, we essentially perform whole-body imaging prior to any intervention. If no other pathologies are noted, we proceed with general surveillance every 3–5 years, usually with magnetic resonance imaging (MRI).

## Conclusion

A right common iliac aneurysm in a 27-month-old was repaired with a cadaveric femoral artery bypass in the setting of limited conduit options. The operation was successful, with continued patency observed at 7 months.

## Data Availability

All data relevant to the case have been included in the manuscript.*

## References

[1] Brunkwall J Hauksson H Bengtsson H , et al. Solitary aneurysms of the iliac arterial system: an estimate of their frequency of occurrence. J Vasc Surg 1989; 10: 381–384. 2795762 10.1067/mva.1989.13733

[2] Iyer H Joharifard S Le-Nguyen A , et al. Microsurgical and endovascular management of congenital iliac aneurysms in the neonatal period: two cases and a literature review. EJVES Vasc Forum 2021; 52: 41–48. 34522908 10.1016/j.ejvsvf.2021.06.007 PMC8424503

[3] Davis FM Eliason JL Ganesh SK , et al. Pediatric nonaortic arterial aneurysms. J Vasc Surg 2016; 63(2): 466–476. 26804218 10.1016/j.jvs.2015.08.099

[4] Falqueto LE Martins GCL Saito AAK , et al. Idiopathic right common iliac artery aneurysm in a three-year-old child - case report. J Vasc Bras 2021; 20: e20200195. 34188670 10.1590/1677-5449.200195 PMC8210647

[5] Sarkar R Coran AG Cilley RE , et al. Arterial aneurysms in children: clinicopathologic classification. J Vasc Surg 1991; 13: 47–56. 1987396

[6] Chithra R Sundar RA Velladuraichi B , et al. Pediatric isolated bilateral iliac aneurysm. J Vasc Surg 2013; 58: 215–216. 23433815 10.1016/j.jvs.2012.11.036

[7] Akturk Y Ozbal Gunes S . Normal abdominal aorta diameter in infants, children and adolescents. Pediatr Int 2018; 60: 455–460. 29498778 10.1111/ped.13542

[8] Zaidan LR Siddique MT Sharif MA , et al. Isolated idiopathic right common iliac artery aneurysm presenting as acute appendicitis in a 9-year-old girl: a case report and literature review. Ann Vasc Surg 2019; 61: e13.469. 10.1016/j.avsg.2019.05.026 31382004

[9] Modrall JG Hocking JA Timaran CH , et al. Late incidence of chronic venous insufficiency after deep vein harvest. J Vasc Surg 2007; 46: 520–525. 17826238 10.1016/j.jvs.2007.04.061

[10] Hartranft CA Noland S Kulwicki A , et al. Cryopreserved saphenous vein graft in infrainguinal bypass. J Vasc Surg 2014; 60: 1291–1296. 24997807 10.1016/j.jvs.2014.05.092

